# Bacterial and archaeal community structures in perennial cave ice

**DOI:** 10.1038/s41598-018-34106-2

**Published:** 2018-10-23

**Authors:** Corina Itcus, Madalina D. Pascu, Paris Lavin, Aurel Perşoiu, Lavinia Iancu, Cristina Purcarea

**Affiliations:** 1 Department of Microbiology, Institute of Biology, Bucharest, Romania; 2 National Institute of Research and Development for Biological Sciences, Bucharest, Romania; 3 Laboratorio de Complejidad Microbiana y Ecología Funcional, Instituto Antofagasta, Universidad de Antofagasta, Antofagasta, Chile; 4 Emil Racovita Institute of Speleology, Cluj-Napoca, Romania; 5 Stable Isotope Laboratory, Stefan cel Mare University, Suceava, Romania

## Abstract

Ice entrenched microcosm represents a vast reservoir of novel species and a proxy for past climate reconstitution. Among glacial ecosystems, ice caves represent one of the scarcely investigated frozen habitats. To characterize the microbial diversity of perennial ice from karst ecosystems, Roche 454 sequencing of 16S rRNA gene amplicons from the underground ice block of Scarisoara Ice Cave (Romania) was applied. The temporal distribution of bacterial and archaeal community structures from newly formed, 400, and 900 years old ice layers was surveyed and analyzed in relation with the age and geochemical composition of the ice substrate. The microbial content of cave ice layers varied from 3.3 10^4^ up to 7.5 10^5^ cells mL^−1^, with 59–78% viability. Pyrosequencing generated 273,102 reads for the five triplicate ice samples, which corresponded to 3,464 operational taxonomic units (OTUs). The distribution of the bacterial phyla in the perennial cave ice varied with age, organic content, and light exposure. Proteobacteria dominated the 1 and 900 years old organic rich ice deposits, while Actinobacteria was mostly found in 900 years old ice strata, and Firmicutes was best represented in 400 years old ice. Cyanobacteria and Chlorobi representatives were identified mainly from the ice block surface samples exposed to sunlight. Archaea was observed only in older ice strata, with a high incidence of Crenarchaeota and Thaumarchaeaota in the 400 years old ice, while Euryarchaeota dominated the 900 years old ice layers, with Methanomicrobia representing the predominant taxa. A large percentage (55.7%) of 16S rRNA gene amplicons corresponded to unidentified OTUs at genus or higher taxa levels, suggesting a greater undiscovered bacterial diversity in this glacial underground habitat. The prokaryotes distribution across the cave ice block revealed the presence of 99 phylotypes specific for different ice layers, in addition to the shared microbial community. Ice geochemistry represented an important factor that explained the microbial taxa distribution in the cave ice block, while the total organic carbon content had a direct impact on the cell density of the ice microcosm. Both bacterial and archaeal community structures appeared to be affected by climate variations during the ice formation, highlighting the cave ice microbiome as a source of putative paleoclimatic biomarkers. This report constitutes the first high-throughput sequencing study of the cave ice microbiome and its distribution across the perennial underground glacier of an alpine ice cave.

## Introduction

Ice habitats, encompassing Arctic and Antarctic ice-sheets, ice shelves and mountain glaciers, unraveled the presence of complex and unique microbiomes^1–5^. In contrast to these icy environments, there is a limited information on the microbial communities from perennial ice accumulations in caves (hereafter “ice caves”)^6^. These ice bodies form throughout the slow freezing of dripping and inflowing water, and/or snow diagenesis. The combination of climatic conditions, cave morphology, and feedback mechanisms^7^ allows for the preservation of ice throughout the year and genesis of millennia-old cave glaciers. The microbial community from such secluded frozen habitats could constitute reservoirs of novel cold-adapted microorganisms^8–10^. Moreover, while perennial cave ice could represent paleoclimatic archives^11–16^, the ongoing climatic changes during cave ice formation are expected to affect the microbial community structure entrapped in various cave ice layers, providing putative biomarkers for the past climate variations. Few ice-contained bacterial species were isolated from Austrian alpine ice caves^17^, and from the rock-ice interface in a lava tube from Oregon Cascades, USA^18^. Three Antarctic fumarolic ice caves near Mt. Erebus were recently explored, and the diversity of the bacterial^19^ and fungal^20^ communities from sub-ice sediments was determined. A recent survey on ice deposits and sediments on Hawaiian lava tubes revealed the presence of a diverse bacterial community and the occurrence of Euryarchaea in such cryogenic formation^21^.

One ice cave study model on ice genesis and dynamics, paleoclimate significance and microbiome of underground perennial ice accumulations is Scarisoara Ice Cave^6,11,15,22–26^. This limestone cave, located at 1165 m asl in the Apuseni Mountains, Romania (46°29′23.64″N, 22°48′37.68″E), hosts the largest (>110,000 m^3^) and oldest (>10,500 years) cave ice block in the world^11,15,22,25–28^. The 22.5 m thick^27^ underground ice block was formed by the freezing of a shallow pond formed on top of the existing ice block, resulting in a structure of alternating layers of clear ice and organic sediment-rich strata^23,29,30^. The origin of the accumulated supraglacial pond is in the drips that reach the cave after percolating through the thin (~30 cm) soil and highly fissured limestone (~15 m thick) above the cave. A small proportion of water also originated from direct precipitation and snow melting at the bottom of the entrance shaft. The ice strata vary between 1–15 cm in thickness^27^, and includes both organic (soil, pollen, plant and animal tissue) and inorganic material derived from the surface and carried inside the cave by both dripping and inflowing water^14,15^. The presence of ice leads to a distinctive underground climate, with air temperatures dropping below 0 °C from late November to early spring (March), and being maintained at 0 °C throughout the remaining of the year, as the heat delivered to the cave by conduction (through the entrance shaft and the rock walls) and dripping water “being consumed” by the slow melting of the ice^30^. The ice block from Scarisoara Ice Cave yielded a wealth of information on the variability of climate and vegetation during the past ~10,000 years^11,15,27^. These studies have shown that during the past ~1,000 years (the timeframe of the present study), climate in the area has changed from warm and wet during the Medieval Warm Period (800–1250 AD) to cold and dry, but with frequent heavy rains, during the Little Ice Age (1250–1850 AD)^27^.

The presence of cultured bacteria was reported from the ice stalagmites of the Little Reservation area of this cave^31^ and from the ice layers up to 900 years old belonging to the perennial ice block^32,33^. Cultured and uncultured fungi were also identified by Denaturating gradient gel electrophoresis (DGGE) analysis in newly formed, 400 and 900 years old cave ice deposits^34^. In this study, we assessed the diversity of uncultured bacterial and archaeal communities across the perennial cave ice block using 454-pyrosequencing, and compared the microbial communities trapped in the cave ice layers of different geochemical signatures formed during various climate-associated periods of the last millennium. This is the first report on the prokaryotic community structure in a cave ice block chronosequence.

## Results

### Microbial cell density in cave ice strata

Total microbial cell density of Scarisoara ice samples measured by flow cytometry (Table 1) ranged from 3.3 × 10^4^ to 7.5 10^5^ cell mL^−1^. The highest cell content (7.5 × 10^5^ cells mL^−1^) was found in the recently formed ice sample 1-S. The cell density decreased with both the age and organic sediment content of the ice deposits. Thus, organic sediment-rich samples 1-S, 400-O (5.1 × 10^5^ cells mL^−1^) and 900-O (1.1 × 10^5^ cells mL^−1^) showed a linear decline of cell content with the age of ice sediment (Supplementary Fig. S1). Meanwhile, both clear ice samples 1-L (2.4 × 10^5^ cells mL^−1^) and 900-I (0.3 × 10^5^ cells mL^−1^) contained 3-fold fewer cells than the corresponding organic-rich ice deposits of same age, indicating a similar age-dependent decline rate of cell density in this habitat. This data confirmed a temporal dependence of microbial cell density in Scarisoara cave perennial ice. Similar tendency of total cell counts and live/dead ratio has been previously observed for the permafrost microcosms formed during the last decade^35^, and 27 kyr to 33 kys BP^36^. The viable cell density of each ice sample (Table 1), calculated as a difference between the total and dead cell contents, showed comparable values in sediment-rich ice strata 1-S (4.4 × 10^5^ cells mL^−1^) and 400-O (3.4 × 10^5^ cells mL^−1^), and 5-fold lower in older ice 900-O (0.8 × 10^5^ cells mL^−1^). The clear-ice samples 1-L (1.9 × 10^5^ cells mL^−1^) and 900-I (0.2 × 10^5^ cells mL^−1^) contained less viable cell as compared to the corresponding sediment-rich strata of the same age, 1-S (2-fold) and 900-O (3-fold), respectively. The cell viability in this habitat ranged from 68–78% for most of the ice layers, with a lower value (59%) for 1-S sample. Active microorganisms, presenting not only basal metabolic functions but also cell growth and division, were found in permanent frozen environments^37–41^. However, limitations of the well-established staining cytometry technique due to the transient permeability of the cell membrane after physical and chemical stress exposures, recently evidenced by Davey and Hexley^42^, suggest that the actual number of viable cells in cave ice deposits could be higher. Such temporary cell membrane damage might occur during ice thawing, aggregate disruption by sonication, and HD-staining processes prior to flow cytometry measurements. Therefore, the lower live/dead cell ratio values obtained for the recently formed light exposed ice layer 1-S could be due to a higher content of less adapted microorganism to life in frozen habitats subjected to thaw stress.

**Table 1:** Cell density of cave ice uncultured microorganisms determined by flow cytometry.

Ice sample	Total cell density (cells mL^−1^) 10^5^	Viable cell density (cells mL^−1^) 10^5^	Viable cells (%)
1-S	7.55 ± 0.68	4.42 ± 0.61	58.6
1-L	2.43 ± 0.20	1.90 ± 0.19	78.2
400-O	5.10 ± 0.60	3.80 ± 0.73	74.5
900-O	1.14 ± 0.03	0.77 ± 0.04	68.2
900-I	0.33 ± 0.01	0.23 ± 0.02	70.6

### Microbial community composition

**Figure 1:**
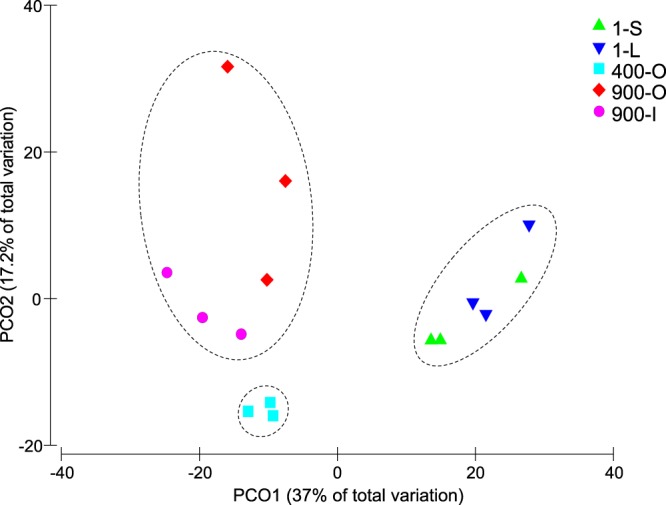
Principal component ordination (PCO) analysis of the prokaryotic OTUs from cave ice. Variation in the OTUs resulted from pyrosequencing data of 1-S, 1-L, 400-O, 900-O and 900-I cave ice samples. Triplicate subsampled OTU libraries at 1,000-bp cutoff were analyzed for each sample (Pseudo-F: 5.0804; p-value = 0.0002 by PERMANOVA).

Quality filtration of the raw pyrosequencing 16S rRNA gene amplicons led to a total number of 273,102 reads for the 15 samples, corresponding to 3,464 OTUs. The number of OTUs found in 1-S, 1-L, 400-O, 900-O and 900-I ice samples varied in the 1,736–2,574 interval, with higher values found in the organic rich samples 400-O and 900-O (Table 2). After subsampling using 1,000-reads cutoff (Table 2), the highest number of observed 16S rDNA OTUs was found in the 400-O (858) and 900-O (737) ice samples, followed by 900-I (596), whereas a lower richness of prokaryotic OTUs characterized the recent ice samples 1-S (256) and 1-L (280). The extrapolated richness (Chao1) and abundance-based coverage estimator (ACE) confirmed this pattern, except for the clear ice sample 900-I that showed the lowest values. The Shannon index variation exhibited a similar trend for all analyzed ice samples (4.00–4.81), with relatively higher values for 400-O and 900-I ice (Table 2). The rarefaction curves of the standardized samples to the smallest library size indicated a partial diversity determined in all ice samples (Supplementary Fig. S2). Principal component ordination (PCO) analysis of the OTUs variation (Fig. 1) showed a clear clustering of the OTUs based on the age of ice, independent of their organic content (900-O and 900-I) and sunlight exposure (1-S and 1-L).

**Table 2:** Number of reads, OTUs, taxon richness and diversity indexes for cave ice samples.

Sample	Number of reads	Total OTUs	Observed OTUs	Estimated OTU richness		Diversity
				Chao1	ACE	Shannon
1-S	11,177 (8,914–15,065)	1736	579	813 (484–1065)	838 (530–1074)	4.20 (2.59–5.48)
1-L	10,825 (6,178–14,546)	1782	594	835 (719–1004)	855 (719–1012)	4.00 (3.60–4.47)
400-O	17,345 (8,419–22,829)	2574	858	1277 (1,098–1412)	1304 (1163–1459)	4.81 (4.35–5.59)
900-O	17,611 (16,151–19,894)	2211	737	1148 (1048–1269)	1182 (1126–1261)	4.42 (3.08–5.61)
900-I	15,341 (13,090–17,942)	1789	596	769 (528–937)	806 (536–667)	4.62 (3.93–5.14)

Subsampled reads, OTUs and statistical paramers values (average, minimum and maximum) are shown for triplicate 1-S, 1-L, 400-O, 900-O and 900-I cave ice samples using 1000-reads cutoff.

**Figure 2:**
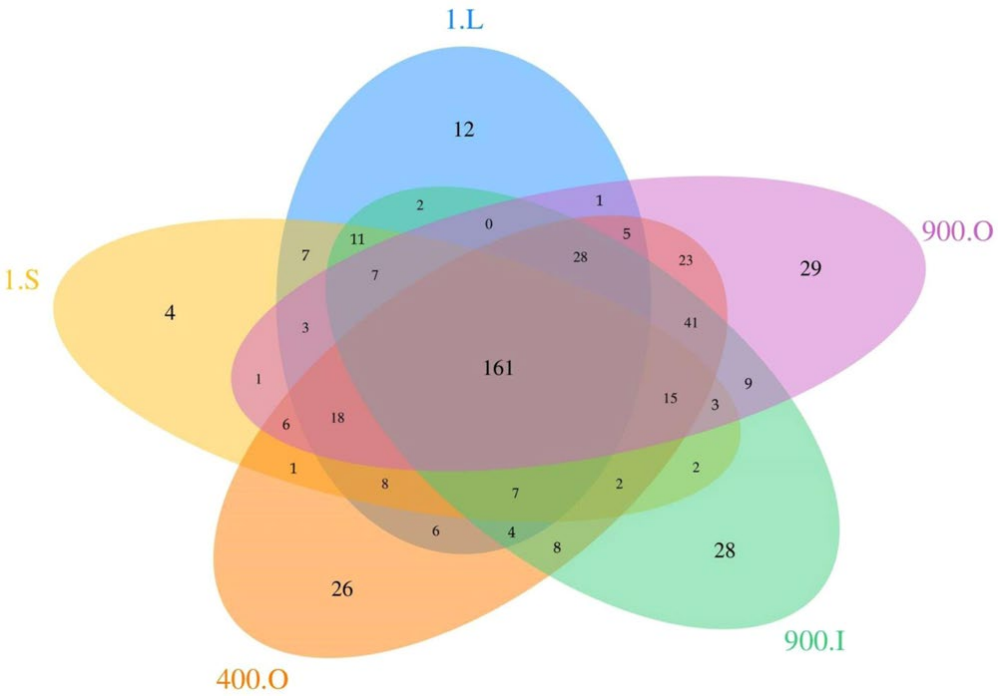
Prokaryotic community distribution in the perennial cave ice block. VENN diagram indicates the number of distinct and shared pyrosequencing OTUs in Scarisoara ice samples 1-S, 1-L, 400-O, 900-O and 900-I.

VENN diagram of the OTUs distribution in the five cave ice layers (Fig. 2) revealed the presence of 99 OTUs unique for each ice sample, representing 20.7% of the total assigned phylotypes (478). Most of the unique taxa were found in older ice deposits 400-O (26), 900-O (29), and 900-I (28), whereas very limited numbers occurred in the recently formed ice 1-L (12) and 1-S (4). The common microbial core comprised 161 distinct OTUs (33.7% of prokaryotic taxa), while 218 OTUs (45.6% of taxa) were shared between different ice layers. Among these, the largest phylotype number (41) was shared between 400 and 900 years old ice, while very few common prokaryotic OTUs were found in both recent ice 1-L (1–6), or 1-S (1–2), and older ice samples 400-O, 900-O and 900-I, respectively.

### Prokaryotes community structure

Bacterial OTUs accounted for 94.9%-99.03% of the ice-contained phylotypes (Table 3), while Archaea was found only in older ice strata, varying from 1.03 ± 1.38% in 400 years old ice, to <1% in both 900 years old ice samples of high and low organic content 900-O (0.24%) and 900-I (0.13%), respectively. Unassigned microbial reads were present in all ice strata, with the highest representation (4.06 ± 1.78%) in 400-O, and low relative content (~1%) in recently formed ice samples 1-S and 1-L.

**Table 3:** Prokaryotic OTUs distribution in ice samples.

Assignment	1-S	1-L	400-O	900-O	900-I
Bacteria (%)	99.03 ± 0.67	98.86 ± 0.38	94.90 ± 3.16	97.33 ± 1.77	98.02 ± 1.24
Archaea (%)	0	0	1.03 ± 1.38	0.13 ± 0.08	0.24 ± 0.17
Unassigned (%)	0.96 ± 0.67	1.13 ± 0.38	4.06 ± 1.78	2.53 ± 1.71	1.73 ± 1.14

**Figure 3:**
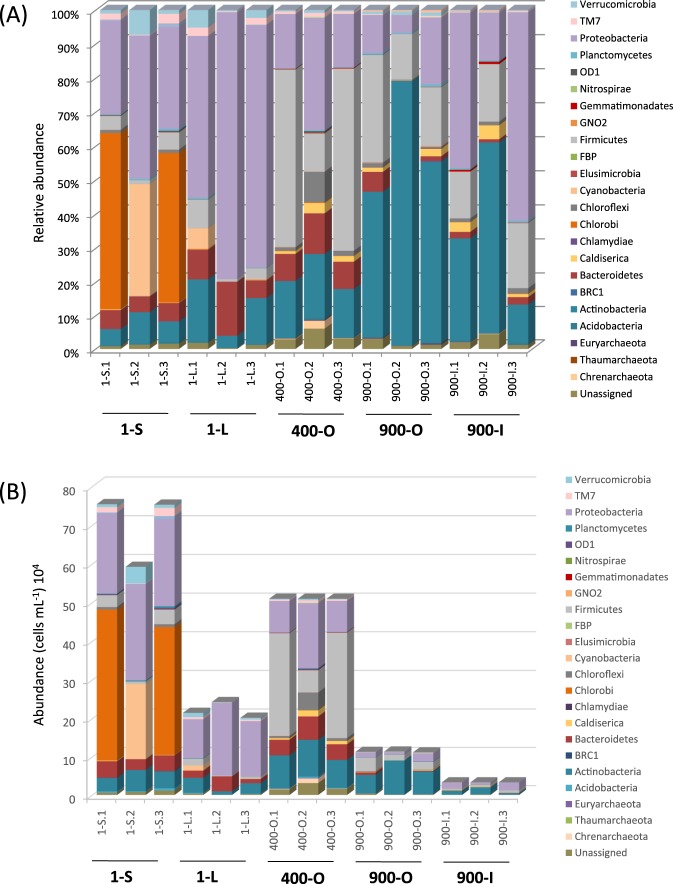
Bacterial distribution in the cave ice sample replicates at phyla level. (**A**) relative bacterial profile; (**B**) deduced quantitative microbial profile taking into consideration the cell density (Table 1) of the cave ice samples 1-S, 1-L, 400-O, 900-O and 900-I.

Identified taxa belonged to 30 prokaryotic phyla. Their distribution in the five cave ice deposits showed strong dissimilarities across the ice block (Fig. 3). The relative abundance profile (Fig. 3A) showed a major incidence of Proteobacteria (33.9%), Actinobacteria (25.6%) and Firmicutes (17.2%). The presence of Proteobacteria (average relative abundance) declined with the age of the ice, while the two most dominant spore-forming phyla, Actinobacteria and Firmicutes, showed an increase in older ice strata. The highest relative content of Proteobacteria was found in the clear ice deposits 1-L (54%) and 900-I (42%). Actinobacteria constituted the major group of the 900-O (up to 54%) and 900-I (39%) ice strata, while only 11% were found in 1-S and 400-O. Firmicutes OTUs were mainly observed in 400-O ice (up to 37% of total taxa). Six other phyla showed a substantial relative abundance (>1%) across the cave ice block. Among these, the phototrophic bacterial phylotypes belonging to Chlorobi (6.7%) and Cyanobacteria (2.2%) were mainly present in the recently formed ice sample 1-S, constituting up to 35%, and 18% of microbial taxa, respectively. Chloroflexi phyla, mostly found in 400-O (5.7%), comprised 1.5% of total prokaryotic taxa. Bacteroidetes OTUs (5.8%) were spread across the ice block, but preferably distributed in 1-L (11%) and 400-O (8.3%) ice layers. Both Caldiserica (1.2%) and Verrucomicrobial (1.4%) OTUs, also identified in all ice layers, were best represented in the 900-I (2.8%) and 1-L (3%) samples, respectively.

The extrapolated quantitative phyla distribution^43^ in different cave ice layers, expressed as the number of mL^−1^ of melted ice, was determined (Fig. 3B; Supplementary Table S1) taking into account the corresponding microbial cell density (Table 1). Thus, the highest Proteobacteria cell density was found in the 1-S (23 × 10^4^ cell mL^−1^), while a 2-fold lower content was present in 1-L and 400-O samples, and 20-fold lower in both 900-O and 900-I samples. Moreover, Actinobacteria, which appeared to dominate the 900-years old strata was highly abundant in the organic rich layers 1-S (4.5 × 10^4^ cell mL^−1^), 400-O (8.5 × 10^4^ cell mL^−1^) and 900-O (6.5 × 10^4^ cell mL^−1^), while the clear ice strata of same age 1-L and 900-I showed a lower cell density by 2-fold and 6-fold, respectively. Firmicutes prevailed in 400-O (27 × 10^4^ cell mL^−1^), in accordance with the highest relative content value for this phylum (Fig. 3A), while only scarcely represented in 1-S and 900-O (10-fold lower abundance), 1-L (30-fold lower abundance), and 900-I (50-fold lower abundance).

Phototrophic bacterial OTUs belonging to Cyanobacteria phylum were mainly identified in the sunlight-exposed recently-formed ice 1-S (55.4%) and 1-L (39.8%) from the ice block surface, while only small fractions were identified in 900-O (4.3%), and in both 400-O and 900-I ice strata 0.2–0.3%) (Supplementary Fig. S3). Chlorobi taxa dominated (99%) 1-S, with only 0.1–0.3% traces in the other ice layers (Supplementary Fig. S3). Quantitatively, both these phyla reached the highest cell density in 1-S sample (Cyanobacteria: 19.5 × 10^4^ cell mL^−1^; Chlorobi: 36.2 × 10^4^ cell mL^−1^), while in 1-L the estimated density of Cyanobacteria was 15-fold lower (Fig. 3B; Supplementary Table S1).

**Figure 4:**
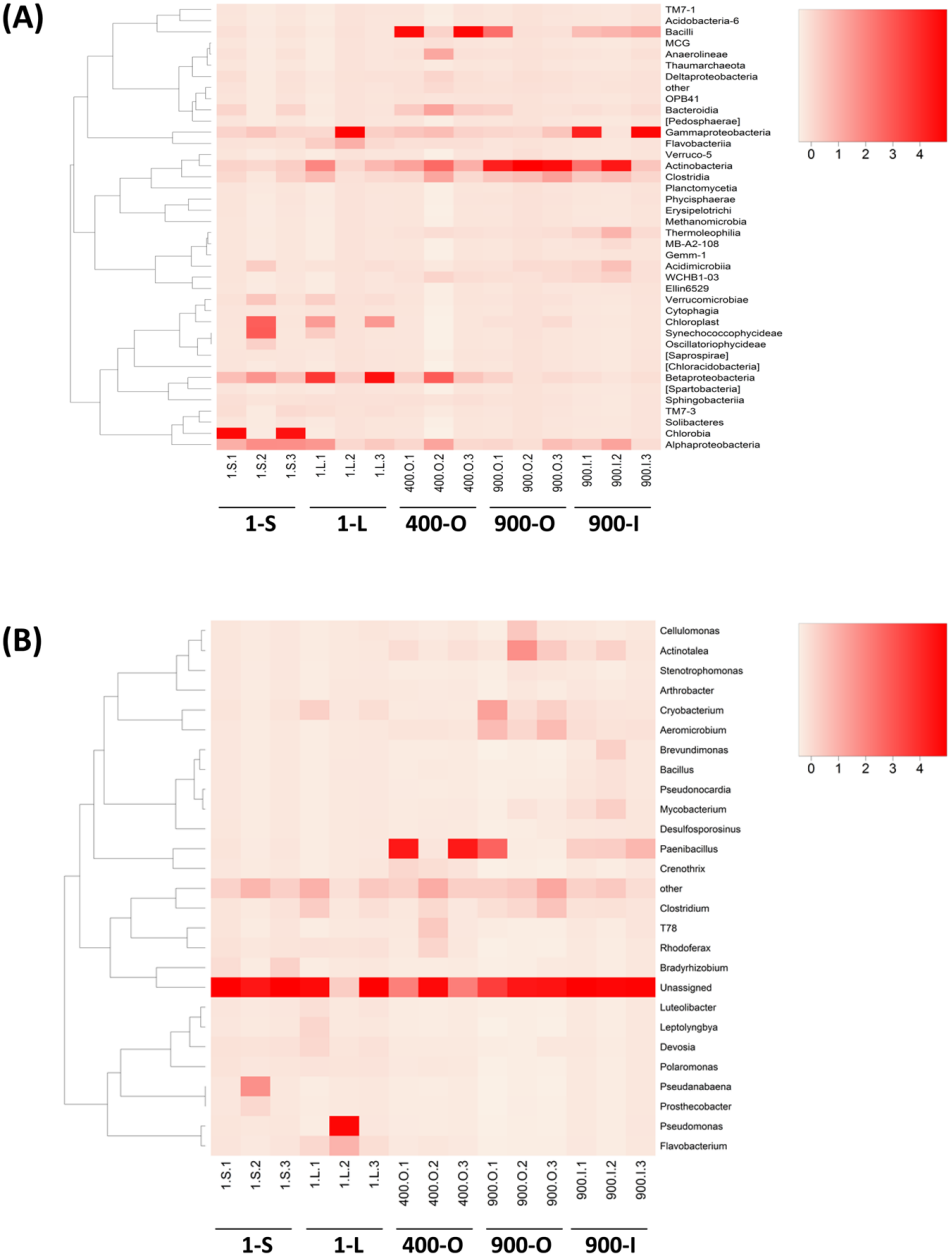
Heat-map analysis of the cave ice prokaryotic community relative abundance at class (**A**), and genus (**B**) levels from 1-S, 1-L, 400-O, 900-O and 900-I replicate samples.

At class level (Fig. 4A), the relative content heatmap revealed the dominance of Gammaproteobacteria in the clear ice samples 1-L and 900-I. Betaproteobacteria was present in 1-S, 1-L and 400-O ice layers, while Alphaproteobacteria was distributed in all aged ice strata. Chlorobia and Synechococcophycideae classes were present in high concentrations in the 1-S ice among phototrophs. Firmicutes was represented by Bacilli class, and was mainly present in the 400-O (6.8%) and 900-I (1.7%) cave ice deposits.

Among the 27 assigned genera of the cave ice microbiome, a high relative content of *Paenibacillus* and *Pseudomonas* was found in 400-O and 1-L samples, respectively (Fig. 4B). *Cryobacterium*, *Aeromicrobium*, and *Actinotalea* species were identified in 900 years old ice, and nitrogen-fixing cyanobacterium *Pseudanabena* spp. appeared in the sun-exposed surface ice 1-S. However, a high percentage of unclassified OTUs at genus level was present in all ice strata (Fig. 4B). Among indigenous bacteria of nonthermal stratified freshwater ecosystems, green nonsulfur T78 bacteria^34^ were present (0.6% relative abundance) in 400-O.

The cave ice phototrophic community comprised 13 cyanobacteria phylotypes. At genus level, *Leptolyngbya*, *Pseudanabaena* and *Phormidium* species were highly represented in 1-S and 900-O ice, while *Acaryochloris*, *Chroococcidiopsis* and *Gloeobacter* had a lower incidence in both 900 years old ice strata. At higher taxa levels, Nostocaceae and Chamaesiphononaceae families, as well as SM1D11 and YS2 orders, were scarcely found in all ice layers. The cave ice microbiome also revealed the presence of three Chlorobi OTUs, dominated by Chlorobiaceae, a green-sulfur bacterial family of obligate anaerobic photoautotrophs^44^. Unidentified Cytophagales/green sulfur bacterium OPB56^45^ and uncultured SJA-28 clade^46^ were scarcely present in both 400 and 900 years old ice strata. In addition, phototrophic purple nonsulfur bacteria belonging to genera Rhodoplanes and Rhodoferax^47,48^ showed a slightly increased relative abundance in the old ice strata, suggesting that bacterial communities from 400 and 900 years old ice were capable of maintaining photosynthetic activities in the presence of very low light intensity^49^.

Moreover, ten Planctomycetes phylotypes belonging to Phycisphaerae, ODP123, Planctomycetia, and vadinHA49 classes were present in all cave ice samples, representing 0.36% of identified prokaryotic taxa. Among these, *Gemmata* and *Planctomyces* OTUs were assigned at genus level. These anammox bacteria that are present in cold environments^50^ occurred mainly in 900 years old cave ice, in accordance with their specific anoxic metabolism.

### Archaeal taxonomy and distribution

**Figure 5:**
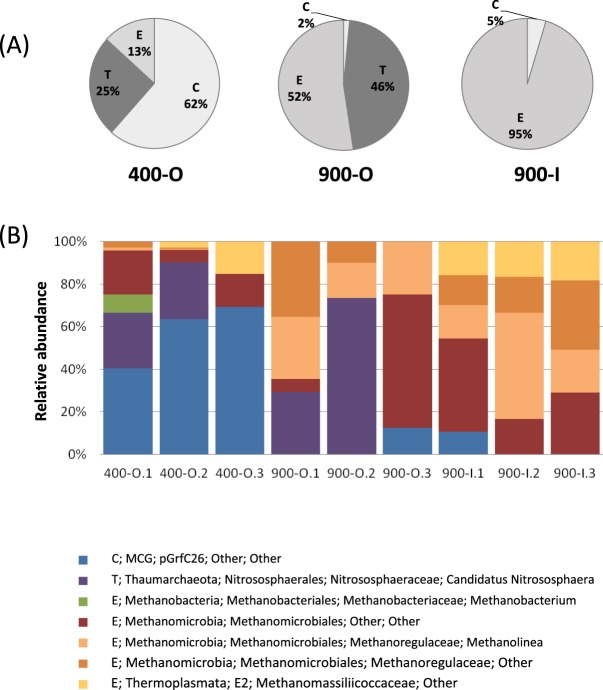
Archaeal community structure in cave ice samples. (**A**) Relative abundance of archaeal phyla C: Chrenarchaeaota; T: Thaumarchaeaota; E: Euryarchaeaota in 400-O, 900-O and 900-I cave ice layers; (**B**) archaeal taxa distribution in the ice sample replicates.

**Figure 6:**
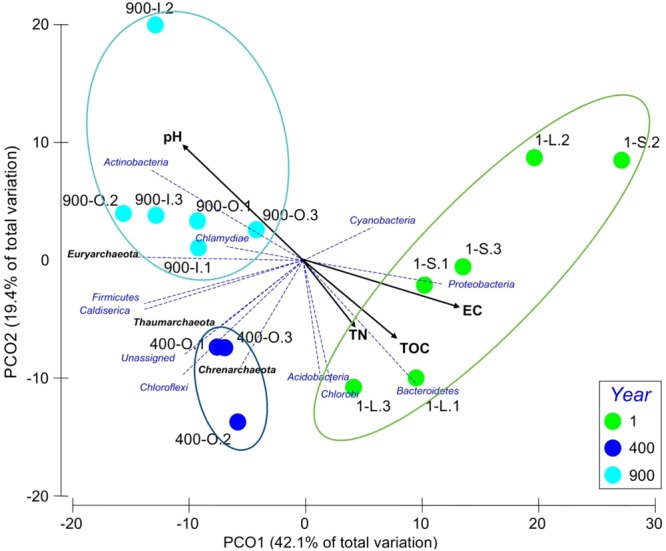
Principle coordinate ordination (PCO) analysis of the cave ice samples in relation with geochemical parameters used to evaluate beta diversity. Colored dots representative of the 3 groups of years samples (Pseudo-F: 5.3847; p-value = 0.0001 by PERMANOVA). The average values of ice pH, electrical conductivity (EC), total organic carbon (TOC), and total nitrogen content (TN), previously determined^32^, and relative content of bacterial and archaeal phyla were used for the analysis of 1-S, 1-L, 400-O, 900-O and 900-I ice sample triplicates. Pearson correlation coefficient (r) values for geochemical data and Phyla varied in the ±(0.494–0.883) and ±(0.122–0.857) interval, respectively.

Archaeal taxa (7 OTUs) were identified only in older cave ice strata 400-O, 900-O and 900-I (Fig. 5). The relative abundance of Crenarchaeota, Thaumarchaeaota and Euryarchaeota phyla was highly variable in the three ice samples (Fig. 6A). Crenarchaeota was the dominant phylum of the 400-O (62% of archaeal taxa) ice layer, while low represented in both 900-O and 900-I ice samples (2–5%). Euryarchaeaota was mostly present in 900-O (52% of archaeal taxa) and 900-I (95% of archaeal taxa), constituting only 13% of the archaeal community of the 400-O ice sample. Thaumarchaeaota was identified in organic-rich ice strata, with the highest occurrence in 900-O (45% of archaeal taxa), and 400-O (25% of archaeal taxa).

The relative abundance of different archaeal phylotypes (Fig. 6B) highlighted a selection of the miscellaneous Crenarchaeota group (MCG) in 400-O ice (40–69%). This ice layer also contained 25% Nitrososphaerales OTUs belonging to the ammonia-oxidizing *Candidatus Nitrososphaera* species, and Methanomicrobiales order OTUs (6–21%), besides a scarce presence of *Methanobacterium* spp. (8%) and Methanomassiliicoccaceae (class Thermoplasmata) (3–15%). Novel *Methanobacterium* species were found in permanently frozen habitats such as Arctic^51^ and Siberian^52^ permafrost. The main archaeal groups in 900-O ice layer were Methanomicrobiales representatives, encompassing *Methanolinea* species (17–29%), and unidentified OTUs at higher taxonomic ranks (42–62%). *Candidatus Nitrososphaera* species belonging to Nitrosphaerales order were present in two replicates with a wide relative abundance variation (29–73%). Meanwhile, the 900-I clear ice was dominated by the strictly carbon dioxide reducing methanogens belonging to Methanomicrobiales (95%), with a high relative content of *Methanolinea* species (16–50%). Representatives of this genus were found in other cold environments including sediments from the permanent frozen lake Fryxell (McMurdo Dry Valleys)^53^ and permafrost from the Tibetan Plateau^54^. Thermoplasmata species were also present in all the 900-I sample replicates, represented by the Methanomassiliicoccaceae family (16–18%).

A statistical ANOSIM analysis indicated a significant difference between the ice samples (*R* = 0.7256, *P* < 0.001).

## Discussion

### Cave-ice microbial proxies for climate variations

This data reporting the bacterial and archaeal abundance and diversity time-span in Scarisoara cave ice strata up to 900 years old represent the first high-resolution record on cave ice microbiome. The spatial and temporal microbial distribution in the perennial ice layers of this cave revealed a heterogeneous prokaryotic community across the ice block. A total of 435 distinct OTUs belonging to bacteria and archaea were identified in recent ice deposits from the ice block surface, 400-years old ice, formed during the Little Ice Age (LIA) period, and 900-years old ice, corresponding to Medieval Warm Period (MWP)^27^. During the last millennium climate was variable, with the MWP experiencing generally warm and wet climatic conditions, while the LIA showed highly changeable hydroclimatic conditions superimposed on a generally cold and dry period^27^. Bacterial communities entrapped in the recently formed ice and in ice deposits accumulated during LIA and MWP periods were dominated by Proteobacteria, Firmicutes and Actinobacteria, respectively. This uneven distribution could be related to the climate variations during ice formation within corresponding temporal periods. Considering the ice formation mechanism in this cave, a major input of cave ice microbiome is expected to be of soil origin. The most abundant phyla from bulk and cold environments’ soils, encompassing Arctic^55,56^, high alpine^57^ and Antarctic^58^ soils, comprise Proteobacteria, Bacteroidetes, Firmicutes and Actinobacteria, their relative abundance depending on the type of carbon source^59^. The predominance of Proteobacteria representatives in the surface layers could be associated with their rapid growth rates on nutrient-rich environments, constituting a proxy for the soil carbon input^60^. Meanwhile, Firmicutes and Actinobacteria are saprophytic organisms that play a critical role in soil decomposition and formation processes^61^. Abundance variation of these phyla in different ice strata could reflect changes in the ratio of obligate aerobes and facultative anaerobes in relation with the carbon source. Hence, the different organic carbon input in LIA and MWP formed cave ice strata could be associated to the shift in Firmicutes and Actinobacteria relative abundances observed in the 400 and 900 years old ice from Scarisoara cave.

The sunlight-exposed ice from the top of the cave ice block (sample 1-S) was characterized by a high content of phototrophs belonging to Chlorobi and Cyanobacteria phyla. The non-specific 16S rRNA gene amplification of algal and plant chloroplast from this sample sustained the high presence of phototrophs in recent ice formed near the cave entrance, as observed in the supraglacial pond formed during summer time in the corresponding small area directly exposed to sunlight^32^.

Specific bacterial and archaeal phylotypes were found in 400 (cold and dry LIA period) and 900 years old (warm and wet MWP period) cave ice samples (Fig. 2), constituting putative biomarkers for climate variations during ice formation. Among the 26 unique taxa inhabiting the LIA-formed ice (400-O), OTUs belonging to genus *Methanobacterium* (Euryarchaeota) revealed the presence of anaerobic hydrogenotrophic methanogens in old ice strata^62^. OTUs belonging to MCG, a predominant archaeal group in anoxic environments, mainly found in deep marine habitats^63^ with possible significant roles in the global biogeochemical cycles, were also highly present (40–69%) in LIA ice layer.

*Koribacter* spp. (Acidobacteria) are among soil acidophiles growing at low temperatures that metabolize carbon monoxide in the presence of high Fe concentration^64^. In Scarisoara cave ice, Firmicutes OTUs belonging to the genus *Dethiobacter* were among the specific 400-O ice microbiota, representing obligate anaerobic chemolithoautotrophs capable of reducing sulphur compounds^65^. This distribution preference is in accordance with the higher iron content of this ice layer as compared to the 900-O (by 1.5-fold) and 900-I ice (4-fold) strata, respectively (to be published elsewhere). The Alphaproteobacteria phylotypes uniquely found in LIA ice layer belonged to the nitrogen-fixing *Pleomorphomonas* genus (Methylocystaceae), while Betaproteobacteria was represented by the denitrifying *Sterolibacterium* species (Rhodocyclaceae). Meanwhile, the ice deposits 900-O and 900-I formed during MWP contained a wide variety of Actinobacteria represented by 10 genera: *Actinomyces*, *Nesterenkonia*, *Pillimelia*, *Nocardia*, *Friedmanniella*, *Actinomadura*, *Williamsia*, *Euzebya*, *Rubrobacter*, and *Patulibacter*. Playing an important role in the soil development and biogeochemical cycling, Actinobacteria survive in extreme environments, being a key component of frozen habitats such as permafrost, glacier forefields, ice cores and cryoconites^4,66^. Moreover, Deltaproteobacteria were mainly found in MWP formed ice, including *Plesiocystis* and the propionate-oxidizing anaerobe *Smithella* species, specific for methanogenic environments^67^.

In comparison with the ice microbiomes from lava caves, our current data revealed a unique bacterial community structure in the perennial ice formed during the last millennium in Scarisoara Ice Cave. Sediments from Antarctic volcanic ice caves in the vicinity of Mt. Erebus Antarctica^19^ indicated a large variation of phyla composition, two of the sites (Harry’s Dream and Hubert’s Nightmare) showing an important relative abundance of Proteobacteria (17–22%). A high representation of Actinobacteria was found in the lava cave characterized by lower temperatures and slightly higher pH, similar to the 900-years old ice layers from Scarisoara Ice Cave. In contrast, Verrucomicrobia phylum, also majorly found (15%) in this Antarctic cave, showed a higher occurrence (7.2%) in the recently formed cave ice sample 1-S characterized by neutral pH. Moreover, Bacteroidetes, representing 22% of Hubert’s Nightmare ice cave sediments, was mostly present in the 1-L and 400-O ice samples. Chloroflexi is one of the dominant taxa (22–66%) in two of the Antarctic caves, but only scarcely represented (1.5%) in Scarisoara ice block where was mostly found in 400 years old ice (8.9%). Meanwhile, a high relative content (26%) of Cyanobacteria populated the light-exposed sediments in Harry’s Dream cave, similar to recently formed ice 1-S from Scarisoara (32%), indicating the flourishing of phototrophic microbiota in various icy habitats exposed to sunlight and their lower conservation in perennial ice after 400 and 900 years. However, while Antarctic caves are not ice caves *sensu stricto* that constitute “rock caves hosting perennial accumulations of ice”^25^, but caves carved in glaciers formed by snow diagenesis, differences in deposition mechanisms most likely have a major impact on the ice-embedded micriobial community structure. Recent investigation of Hawaiian lava tubes^21^ highlighted the presence of various bacterial and archaeal communities in silica and calcite deposits and ice pond water. The ice bacterial community was dominated by Proteobacteria phylum (39%), similar to the recent ice deposits of Scarisoara Ice Cave, in addition to Bacteroidetes (18%), Verrucomicrobia (8%) and candidate division OD1 (7%) also spread in the 1-S, 1-L and 400-O alpine cave ice samples. Meanwhile, the mineral samples of Hawaiian volcanic caves had a high relative abundance (86%) of Actinobacteria, similar to 900-years old ice layers of the alpine ice block, and Euryarchaeota OTUs were also found in the volcanic ice cave, but very scarcely represented (<1%) unlike the MWP-formed ice layers of Scarisoara cave. However, in spite of some commonly found taxa in these two types of caves, a wider diversity and a particular composition and distribution were characterizing the prokaryotic community across the perennial ice block of Scarisoara Ice Cave. This could be partly due to the less abundant soil cover above the Hawaiian caves, as compared to that above Scarisoara cave, where a 20–50 cm thick podosol developed under mixed spruce and birch forest mantles the cap rock above the cave.

In addition, ice deposition process (thickness of ice strata) could be influenced by climate variations during ice formation, the environmental parameters (temperature and precipitation) being in direct relation with the thickness of the ice strata, thus affecting the microbial cell density in successively formed ice strata. Taking into consideration that the temperature in the glacial part of the cave showed little variations around 0 °C^30^, evaporation of the formed supraglacial lake prior to annual freezing is not expected to play a decisive role in ice layer thickness. Therefore, ice layers formed during the dryer LIA period (sample 400-O) showed a higher (by 3.5-fold) microbial cell density relative to that of the wetter MWP period (sample 900-O), in accordance with differences in the precipitation regime. Such correlation was also observed in the glacier ice cores from Tibetan Plateau, where a higher microbial abundance was reported for dryer regions, while lower abundance occurred in areas with high precipitations affected by the Indian Monsoon^35^. Also, Antarctic ice core formed during the last 68 kyr showed higher cell density in strata formed during the colder and dryer Last Glacial Maximum period^68^.

Meanwhile, the prokaryotic diversity (Shannon index) from the ice cave showed only a 10% decrease between LIA and MWP formed ice, unlike the lower prokaryotic variability during dryer periods in both Tibetan and Polar glacier ice cores^35,68^, suggesting a more complex input of various modeling factors of the microbial community structure in Scarisoara cave ice block.

While the ice block was mainly formed from freezing of inflowing and dripping water from the cave surface^14,15^, the source of microorganisms in the perennial cave ice is expected to be mainly the surrounding soils and karst/ground water. In Scarisoara ice, the pyrosequencing data showed that the most abundant prokaryotic taxa from each ice sample (Supplementary Table S2) originated from soil/rhizosphere, water, glacier ice and sediments, with a high content of bacterial homologues from various icy habitats.

### Ice geochemistry impact on bacterial variability and abundance

The geochemistry of the 1-S, 1-L, 400-O, 900-O and 900-I cell-free melted ice samples (using 0.22 µm membranes) was previously determined (Supplementary Table S3)^33^. Neutral to slight alkaline pH (7.45–8.03) was found across the ice block, with higher values in 900-years old ice strata formed during MWP interval as compared to that of LIA-formed ice. Electrical conductivity (EC) varied in from 15 µS cm^−1^ to 124.2 µS cm^−1^, showing a decrease with the age of ice. Total organic carbon (TOC) content ranged in the 3.03–33.38-mg L^−1^ interval, with higher values for 1-S (33.38 mg L^−1^) and 400-O (29.96 mg L^−1^) samples, and reduced concentrations for the recently formed clear ice and 900 years old ice samples by 3-fold (1-L), 5-fold (900-O) and 10-fold (900-I), respectively. The highest total nitrogen (TN) concentrations were also found in 1-S and 400-O samples (2.15–2.23 mg L^−1^), while clear ice 1-L (0.53 mg L^−1^) and both 900 years old ice samples 900-O and 900-I (0.62–0.64 mg L^−1^) showed reduced nitrogen content by 4 and 5 fold, respectively.

A PCO analysis of the microbial phyla composition from all investigated ice deposits in relation with the ice geochemical parameters (Fig. 6), explained 61.5% of the data variation for the separation on PCO1 and PCO2 axes.

The observed distribution of bacteria and archaea in the cave ice block, based on the taxonomic assignment at phylum level (Figs 4 and 6), was confirmed by the β-diversity analysis of the microbial community structure. Considering Bray–Curtis dissimilarities, a PCO analysis (Fig. 6) showed a clear difference between samples from different age, confirming the dissimilar microbial community structure entrapped in 1, 400 and 900 years old ice (PERMANOVA analysis; Pseudo-F: 5.3847; p-value = 0.0001 by PERMANOVA). The distribution of microbial phyla in 1 year old ice was closer to the 400 years old population, while that of 400-O samples was more similar to the microbial communities formed in 900 years old ice (900-O and 900-I samples) (Fig. 6), suggesting a progressive change of populations, as an effect of the geochemistry substrate transition on the ice layers. Thus, the pH had large positive loadings, while EC and TOC had negative loadings on PCO1, with about equal contribution to sample partition. The 400-O ice microbial community showed a high score on PCO2, well separated from the other samples based on the TN ice content, with a strong correlation of Chloroflexi, Firmicutes and unassigned phyla distribution. The relative content of Cyanobacteria and Proteobacteria from recently formed ice 1-S and 1-L was grouped based on EC. The content of Actinobacteria in old ice strata 900-O and 900-I appeared to be strongly dependent on the pH of the ice substrate (Fig. 6).

Moreover, both the total organic carbon content appeared to model the prokaryotic cell concentration in different ice strata, with a good correlation between TOC and cell abundance (Table 1, Supplementary Table S3). A 5-fold decrease of both cell density and TOC content was observed between 400-O and 900-O samples. This strict correlation is in accordance with the precipitation regime during LIA and MWP, affecting the concentration of total organic carbon and microbial communities from the supraglacial pond accumulated in the cave by the same factor. Moreover, in the case of clear ice formed during MWP (900-I) relative to that of 400-O, the higher decrease of cell density (by 15-fold) relative to that of TOC (by 10-fold) suggests the impact of organic carbon content during deposition on the cell abundance in this habitat, favoring the development of a more abundant microbial community in the water lake accumulated on top of the cave ice block prior to freezing, and its contribution in post-depositional processes for sustaining an active prokaryotic community.

In addition, the carbon content of ice appeared to also affect the variability of prokaryotic community from Scarisoara cave ice block. For the same aged ice, the organic rich layer 900-O showed a higher bacterial diversity (20% more observed bacterial OTUs) than the corresponding clear ice layer 900-I. Also, the MWP formed ice (900-O) contained 14% less OTUs than the LIA-formed ice layer (400-O), partially correlated with the decrease in TOC (by 20%) between these ice strata.

The post-depositional processes following the formation of ice layers could also be considered as a modeling factor of the cave ice bacterial profile^69^. The constant below-freezing temperature (<0 °C) in the vicinity of the ice block from Scarisoara cave^30^ could favor metabolic activity in the formed ice liquid-water veins, as reported for temperate glacier ice^70^. Thus, the putative cell division occurring in the cave ice for the last millennium could have an impact on the cell abundance and community structure of recently-formed and older ice strata, considering the neutral to slightly alkaline pH of all ice samples, suitable for active bacteria occurrence^71,72^. Taking into account the total and viable microbial cell density from each ice strata (Table 1) and the corresponding ice TOC content (Supplementary Table S3), the estimated metabolic rates of the ice microbial community^69^ were calculated based on the variation of organic carbon content per microbial cell entrapped during 500 years between 400 and 900 years old ice strata, and considering the average carbon mass per cell of 86 fg, specific for terrestrial aquifers^73^. The theoretical rates, expressed as grams of carbon incorporated into cell material per gram of total biomass carbon per hour^69^, for clear ice (900-I) microbiome were of 1.5 × 10^−5^ g C (g C)^−1^ h^−1^ (total biomass) and 2.2 × 10^−5^ g C (g C)^−1^ h^−1^ (viable biomass), while no metabolism (total biomass) or very reduced (5.7 × 10^−7^ g C (g C)^−1^ h^−1^ for viable biomass) was estimated for the microbiome within the 900-O organic rich ice layer. At temperatures around freezing point (0 °C) that characterize Scarisoara cave ice block^30^, these values correspond to metabolic rates for maintenance of cellular functions in clear-ice (900-I), and for survival in organic rich ice (900-O), based on Price and Sowers study on temperature-dependence metabolic rates of microbiomes from various cold environments^69^. In this case, differences between metabolic rates could be explained by the presence of a more abundant heterotrophic community in organic-rich strata (400-O and 900-O) as compared to that from clear ice (900-I), thus recycling organic carbon at a higher rate. However, these hypothetical metabolic rates sustaining the presence of active microbial cells in this habitat remain to be confirmed by determining the actual microbial contribution to the ice carbon content through direct measurements of the microbiome activity. Meanwhile, active bacteria presence across a 25-m deep ice core from Scarisoara cave was confirmed, based on Illumina sequencing of RNA transcripts (to be published elsewhere), sustaining the contribution of post-depositional processes that are modeling the bacterial community structure across the cave ice block.

The current data on Scarisoara Ice Cave microbiome underscored a highly diverse bacterial community throughout the cave ice block, and the occurrence of various archaeal taxa in old ice layers formed during 400 and 900 years BP, revealing large dissimilarities in the ice-embedded microbial profiles that varied with the age, organic content and light exposure of the ice. Both heterotrophic and autotrophic prokaryotes were identified in recent and old ice deposits, with the putative presence of various anaerobic microbial groups. In addition, climate variations recorded during ice formation within LIA and MWP time-intervals appeared to have an impact on both major bacterial and archaeal phyla, with distinct prokaryotes identified up to genera level that might serve as putative climate proxies. Extended investigations on the total and active microbial communities from Scarisoara cave ice block accumulated during the last 13,000 years, currently underway, will contribute to unravel the role of cave ice microbiome in shaping this frozen habitat in relation with the substrate geochemistry and climate variations, and to provide novel psychrophilic strains of high applicative potential in bionanotechnologies.

## Material and Methods

### Ice samples

**Figure 7:**
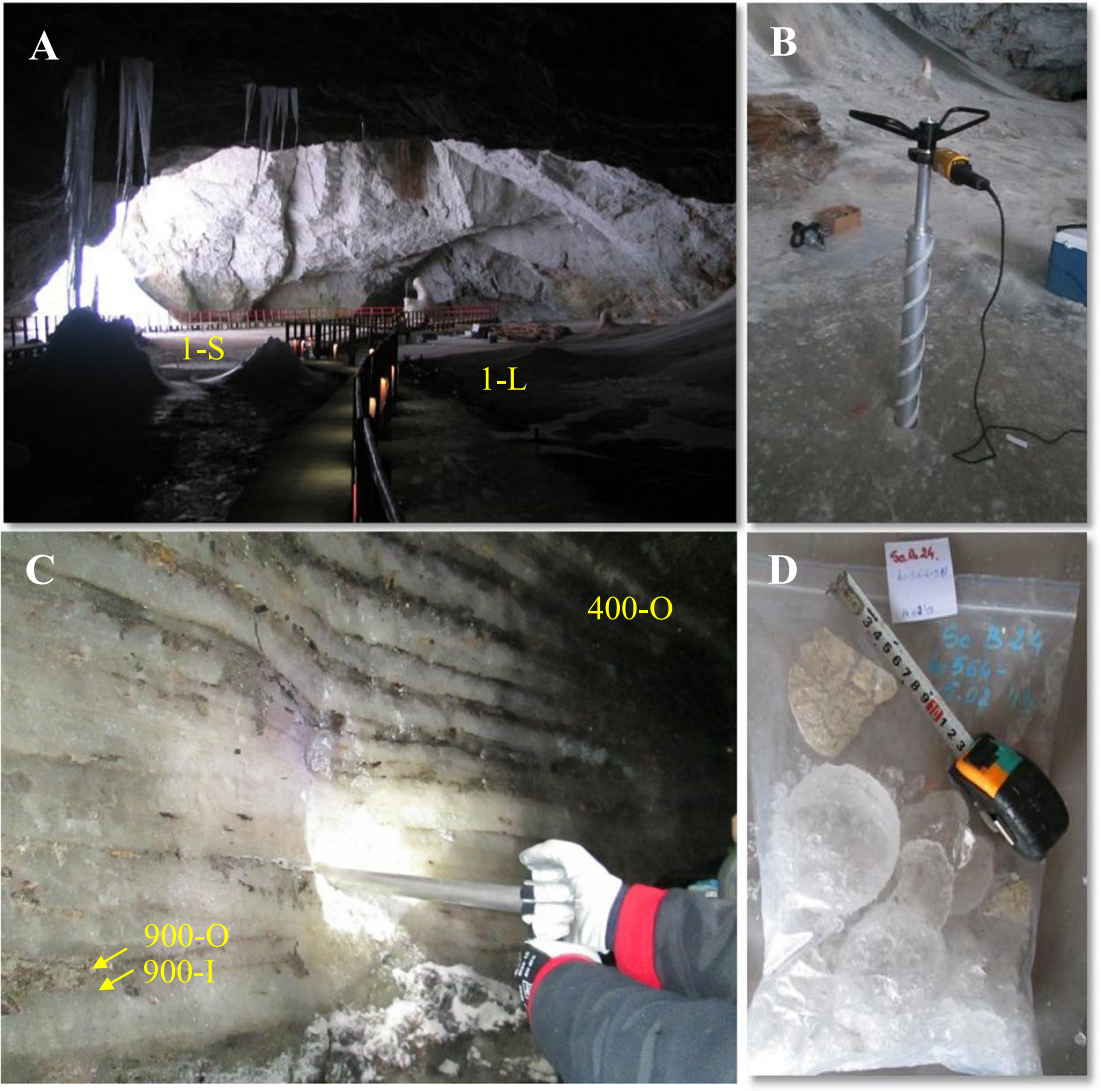
Sampling locations in Scarisoara Ice Cave. (**A**) Samples 1-S and 1-L collected from the Great Hall (photo C. Purcarea); (**B**) Vertical drilling on the top of the ice block (photo C. Itcus); (**C**) Samples 400-O, 900-O and 900-I collected by horizontal drilling in the Little Reservation. O: organic ice sediment; I: clear ice (photo M.D. Pascu); (**D**) Ice core fragments (photo M.D. Pascu).

Cave ice was sampled from different layers of the perennial ice block located in Scarisoara Ice Cave, corresponding to five sample ^14^C-dated to 1-year old, 400-years old and 900-years old ice deposits^15,33^. The 400 and 900 years old ice layers correspond to Little Ice Age (LIA) and Medieval Ice Period (MWP) climate periods, respectively. Recent ice samples 1-S and 1-L were collected from direct (1-S) and indirect (1-L) sunlight-exposed areas of the ice block surface located in the *Great Hall* zone of the cave (Fig. 7A). Sampling was carried out by vertical drilling, using an electrical 10-cm diameter ice corer (Fig. 7B). The 400-O, 900-O and 900-I ice samples were collected from high (O) and low (I) visible organic sediment-rich ice layers^27,32,33^ (Fig. 7C), confirmed by total organic carbon (TOC) content analysis^32^, from the *Little Reservation* exposed ice wall. After removing a 20-cm surface ice layer, sampling was carried out by horizontal drilling. To avoid contamination, the inner and outer surfaces of the drill were treated with 96% ethanol and flamed after each step. Ice samples (Fig. 7D) directly placed in sterile flaks or plastic bags were transported in a continuously frozen state using Coleman 100QT Xtream cool boxes, and stored at −20 °C.

### Flow cytometry

The cell density of total and viable microorganisms contained in ice samples was measured by flow cytometry, using a Gallios flow cytometer (Beckman Coulter, Wien, Austria). Ice samples (1 ml) were thawed at 4 °C under sterile conditions, and the cellular aggregates were dispersed in the presence of 0.1% Tween 80 (Sigma-Aldrich, Munich, Germany) after two cycles of incubation for 10 min in an ultrasonic bath Sonorex Digital 10 P (Bandelin, Berlin, Germany) at 5% power, followed by 1 min vortexing. The resulted suspension was labeled with 1 × SybrGreen I (SG) (Lonza Group, Basel, Switzerland) for total cell counting^74^ and with the cell-impermeable viability marker ethidium homodimer-1 (HD) (ThermoFisher Scientific, Darmstadt, Germany) (1 × final concentration) to determine the live/dead cell content^75^. The number of total and dead cells contained in 90 μl treated ice sample was measured immediately after labeling. The density of living cells was calculated by subtracting the number of HD-labeled (dead) cells from the total number of SG-labeled microorganisms. Cell density was expressed as number of cells contained in 1 ml of melted ice. Unstained cells were used as negative controls.

### 16S rRNA gene amplification and 454 pyrosequencing

Microbial cells were collected from melted ice samples (500 ml) at 4 °C by filtration on 0.22 µm MF-Millipore sterile membrane filters (Merck Millipore, Darmstadt, Germany). Before melting, ice samples were subjected to superficial layer removal (~1 cm) to eliminate possible contamination. Genomic DNA was extracted using DNeasy Blood and Tissue kit (Qiagen, Hilden, Germany) by a modified protocol in the presence of mutanolysin^33^. All these steps were carried out in a Class II Type A2 ASAL900 Atlantic laminar flow hood (ASAL S.R.L., Italy), under aseptic conditions, to avoid contamination. 16S rRNA gene libraries of ice microbiota were obtained in triplicate by 454 pyrosequencing for the selected ice samples. A first amplification step was carried out in 20 μl mixture containing 5 x *Phusion* HF buffer (ThermoFisher Scientific, Darmstadt, Germany), 0.2 mM dNTP mixture, 0.4 U *Phusion* Hot Start II DNA Polymerase (ThermoFisher Scientific, Darmstadt, Germany), 0.5 µM of 341 F (5′-CCTACGGGAGGCAGCAG-3′) and 806 R (5′-GGACTACYVGGGTATCTAAT-3′) prokaryotic primers flanking the V3-V4 hypervariable regions^76^, and 1 µl of 10 x diluted DNA template (11 ng). PCR amplification started with incubation at 98 °C for 30 s, followed by 30 cycles of 98 °C for 5 s, 56 °C for 20 s and 72 °C for 20 s, with a final extension at 72 °C for 5 min. The PCR products were migrated on a 1.25% (w/v) agarose gel, extracted and purified using Montage DNA Gel Extraction Kit (Merck Millipore, Darmstadt, Germany). In a second step, 1 µl of purified PCR-product was amplified using the reaction mixture of the previous PCR step in the presence of MID-barcoded 341F and 806R primers, a generous gift from Dr. Carsten S. Jacobsen (Geological Survey of Denmark and Greenland, Department of Geochemistry, Copenhagen, Denmark), by following the same reaction protocol but with a reduced number (15) of cycles. The amplicons were migrated and purified as described for the first amplification step, and the DNA fragments were quantified using a Qubit fluorometer (ThermoFisher Scientific, Darmstadt, Germany). Equal amounts (100 ng) of each DNA sample were mixed up, and the 454 pyrosequencing of the pooled amplicons was carried out on a Genome Sequencer FLX Titanium Pico TiterPlate using a GS FLX Titanium Sequencing Kit XLR70 (Beckman Coulter Genomics, USA).

### Data analysis

The raw pyrosequencing dataset was analyzed (MBioinform SA, Copenhagen, Denmark) using QIIME^77^. Demultiplexing of the reads using the Roche 454 GS FLX Titanium platform implied a minimum and maximum read length of 200 and 1,000 bp, respectively, allowing 6 ambiguous bases per read, with a minimum mean Phred quality score of 25. After sequence denoising using denoise_wrapper.py software^78^, the OTU selection was carried out by USEARCH clustering method^79^ at 97% similarity for removing chimeras and singletons^80^, against the Greengenes 13_08 reference database^81,82^. Sequences were aligned with PyNAST^83^ against the QIIME core set of aligned sequences, and the taxonomy was determined with UCLUST consensus taxonomy assigner^77^. The read counts were rarefied to equal depth to the level of the sample with the lowest reads. Statistical analysis using ANOSIM^84^ was also conducted in QIIME.

Diversity analyses were carried out using R package vegan: Community Ecology package^85^. Beta Diversity in ice samples was examined using permutational multivariate analyses of variance (PERMANOVA)^86^. PERMANOVA was conducted using model with Type III sum of squares and 9999 permutations on resemblance matrix constructed using a Bray Curtis similarity measure for Phyla composition, on fourth-root transformed. Principal coordinate analysis (PCO)^86^ performed on the resemblance matrix was used to visualize structure within different years of ice samples, with vectors for the geochemical data and Phyla that were correlated (Pearson’s r) with the ordination structure. The PCO biplot was generated using PRIMER software, by overlapping the separately generated plots for environmental and phyla data. PERMANOVA analyses and ordinations were made using PRIMER (version 6.1.16). The VENN diagram was generated using the R free environment for statistical computing and graphics software^87^.

## Electronic supplementary material

Supplementary material

## Data Availability

Raw pyrosequencing reads were deposited in the NCBI Sequence Read Archive under the BioProject accession number PRJNA305850, corresponding to BioSample accession numbers of triplicate samples SAMN04334770, SAMN04334771, SAMN04334772 (sample 1-S), SAMN04334767, SAMN04334768, SAMN04334769 (sample 1-L), SAMN04334773, SAMN04334774, SAMN04334775 (sample 400-O), SAMN04334776, SAMN04334777, SAMN04334778 (sample 900-O), and SAMN04334790, SAMN04334791, SAMN04334792 (sample 900-I).
