# Supplementary material for: Bacterial and archaeal community structures in perennial cave ice
Source: Sci Rep. 2018 Oct 23;8:15671. doi: 10.1038/s41598-018-34106-2 (PMC6199274; doi:10.1038/s41598-018-34106-2)
Supplement: Supplementary file 1 — Supplementary material [file 41598_2018_34106_MOESM1_ESM.pdf]

SUPPLEMENTARY MATERIAL

Bacterial and archaeal community structures in perennial cave ice

Corina Itcus[1,2#], Madalina D. Pascu[1#], Paris Lavin[3], Aurel Perşoiu[1,4,5], Lavinia Iancu[1], Cristina Purcarea[1*]

[1] Department of Microbiology, Institute of Biology, Bucharest, Romania

[2]National Institute of Research and Development for Biological Sciences, Bucharest, Romania

[3]Laboratorio de Complejidad Microbiana y Ecología Funcional, Instituto Antofagasta, Universidad de Antofagasta, Antofagasta, Chile

[4] Emil Racovita Institute of Speleology, Cluj-Napoca, Romania

[5] Stable Isotope Laboratory, Stefan cel Mare University, Suceava, Romania

Keywords: ice cave, diversity, bacteria, archaea, 16S rRNA gene, pyrosequencing, perennial ice

*** Corresponding author:** cristina.purcarea@ibiol.ro

#Authors with equal contribution to this work

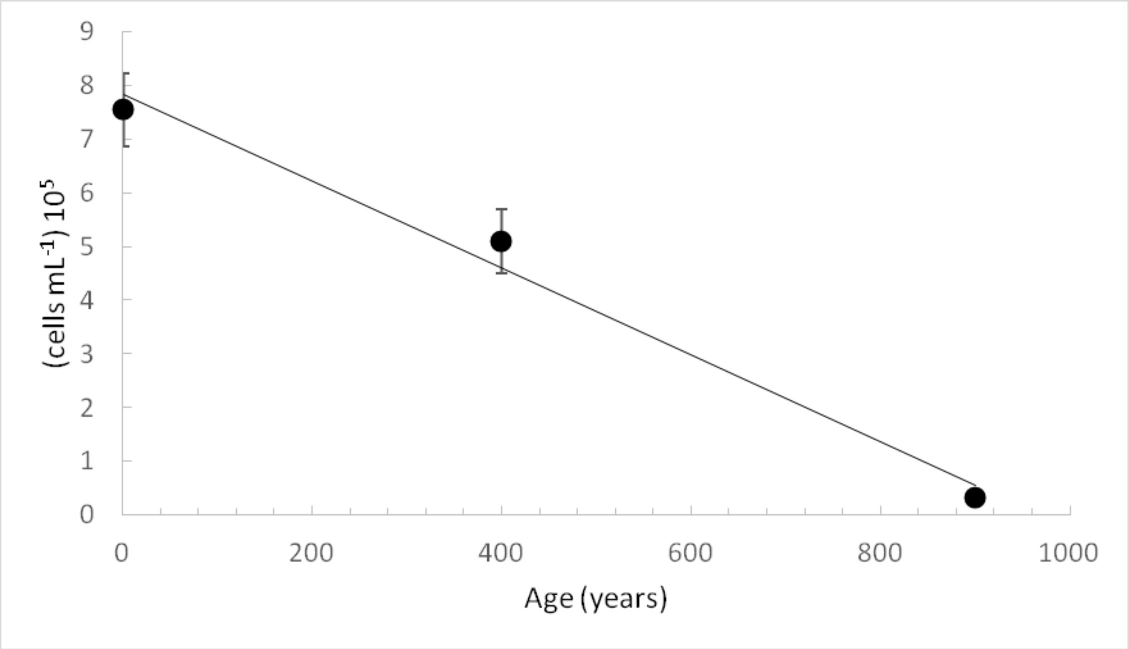

Supplementary Figure S1. Variation of microbial content with the age of ice. The average and standard deviation values of the cell content from 1-year old ice (1-S and 1-L), 400 years old ice (400-O) and 900 years old ice (900-O and 900-I) determined by flow cytometry were plotted against the age of corresponding ice layers.

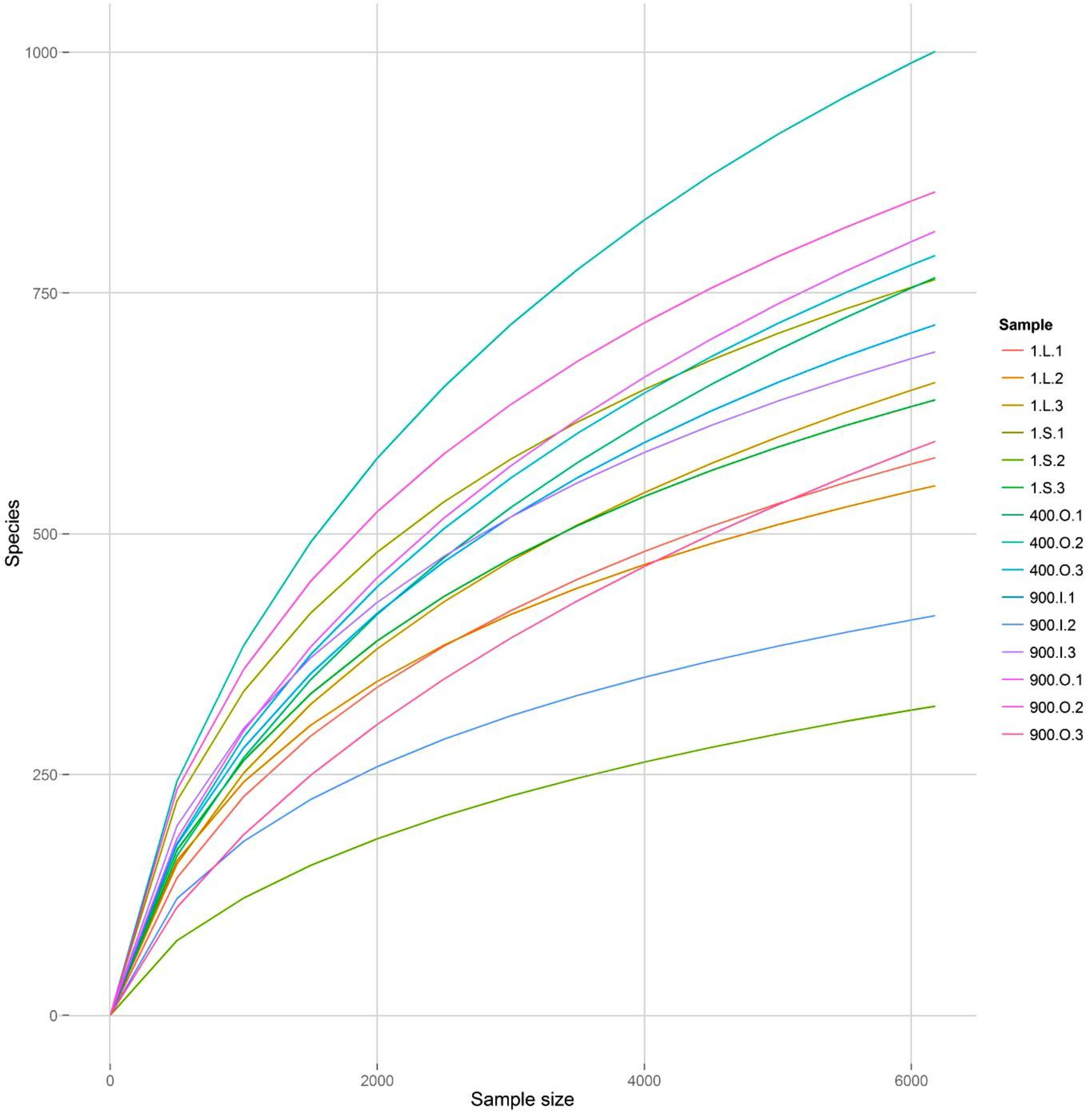

Supplementary Figure S2. Rarefaction curves obtained from pyrosequencing data of 1-S, 1-L, 400-O, 900-O and 900-I ice samples, in triplicate. The plot indicates the number of reads obtained for each sample considering the minimum number of counts (6,178) for all samples.

Supplementary Table S1. Estimated microbial abundance at phyla level calculated by multiplying the relative phyla content with the cell content (Table 1) measured for Scarisoara ice samples.

Phyla	Estimated cell abundance (cell mL^{-1}) x 10^4														
	1-S.1	1-S.2	1-S.3	1-L.1	1-L.2	1-L.3	400-O.1	400-O.2	400-O.3	900-O.1	900-O.2	900-O.3	900-I.1	900-I.2	900-I.3
**Unassigned**	0.62326	0.770998	1.178228	0.392551	0.06615	0.245178	1.478908	3.120947	1.617413	0.346282	0.102765	0.145247	0.063863	0.147316	0.039508
**Chrenarchaeota**	0	0	0	0	0	0	0.107232	1.209612	0.05452	0.003812	0	0	0.001659	0.004495	0.000197
**Thaumarchaeota**	0	0	0	0	0	0	0	0	0	0	0	0	0	0	0
**Euryarchaeota**	0	0	0	0	0	0	0.053616	0.130039	0.024231	0.032404	0.005225	0.041825	0.003981	0.001635	0.001376
**Acidobacteria**	0.31774	0.19275	0.560566	0.155385	0.01781	0.072585	0.071488	0.333686	0.163559	0.00826	0.006096	0.0327	0.017583	0.00756	0.022801
**Actinobacteria**	3.519586	5.570462	4.437818	3.922784	0.875217	2.772765	8.663454	9.637641	7.311676	4.858756	8.883988	5.939924	0.997929	1.852789	0.375424
**BRC1**	0.048883	0	0.062285	0	0	0	0.017872	0.019629	0.012115	0.012072	0.013934	0.035741	0.000166	0.000409	0.000786
**Bacteroidetes**	4.313937	2.814143	4.162725	1.878248	3.933389	1.056522	4.070349	6.089772	4.101081	0.657619	0.006096	0.161217	0.0632	0.030444	0.073709
**Caldiserica**	0.183312	0.01285	0.098618	0.010904	0.005088	0.008065	0.411056	1.550659	0.854139	0.142325	0.013934	0.240304	0.09339	0.135261	0.030859
**Chlamydiae**	0	0	0.00519	0	0	0.001613	0	0	0	0.009531	0	0.008365	0.000332	0.000613	0
**Chlorobi**	39.16761	0.006425	33.17204	0.035439	0	0.027421	0.049148	0.088329	0.036346	0.003177	0	0.007605	0.002322	0.00143	0.004717
**Chloroflexi**	0.672143	0.044975	0.695518	0.005452	0.002544	0.012904	0.549564	4.558741	0.714812	0.13343	0.018289	0.062357	0.034171	0.03453	0.05366
**Cyanobacteria**	0.012221	19.53195	0.015571	1.308503	0	0.041938	0.062552	0.024536	0	0.005083	0.003484	0.034221	0.000332	0.001022	0
**Elusimicrobia**	0.024442	0	0.041523	0	0	0	0.002234	0.014721	0.012115	0.010801	0	0	0	0	0
**FBP**	0	0.019275	0	0.002726	0	0	0	0.002454	0	0	0	0	0	0	0
**Fibrobacteres**	0	0	0	0.005452	0	0	0.006702	0.009814	0.018173	0	0	0.003802	0.000332	0	0
**Firmicutes**	3.042975	0.610374	3.757872	1.799192	0.16792	0.635526	26.53099	5.702107	27.30823	3.574652	1.516226	1.907224	0.451523	0.555755	0.630949
**GNO2**	0	0	0.010381	0.002726	0	0	0.020106	0.017175	0.012115	0	0	0.002281	0.001327	0	0.000393
**Gemmatimonadates**	0	0	0.015571	0.005452	0	0	0.053616	0.071154	0.084808	0	0.000871	0.006084	0.01692	0.022271	0.003145
**Lentisphaerae**	0.061104	0	0.083047	0	0	0.003226	0.006702	0.014721	0	0	0.000871	0	0.000332	0.001226	0.00059
**Nitrospirae**	0.012221	0.01285	0	0	0	0	0	0.019629	0	0	0	0	0.000498	0.000409	0.00059
**OD1**	0.15887	0	0.295855	0.01363	0	0.008065	0.04468	0.225729	0.036346	0.010166	0	0.003802	0	0.001635	0
**Planctomycetes**	0.171091	0.256999	0.425615	0.092686	0	0.035486	0.040212	0.176657	0.048462	0.050195	0.044416	0.08289	0.016422	0.011238	0.018869
**Proteobacteria**	20.70201	24.96749	22.58356	10.1927	19.0258	14.37192	8.1474	16.79962	8.002257	1.265043	0.590466	2.18403	1.50668	0.462176	2.020216
**SR1**	0.293299	0.019275	0.752612	0.005452	0.002544	0.035486	0.006702	0.019629	0.006058	0.003812	0	0	0	0	0
**Spirochaetes**	0	0	0.025952	0	0	0	0.004468	0.002454	0	0	0.007838	0.003802	0	0	0
**TM6**	0	0	0	0.002726	0	0.012904	0.020106	0.147214	0.036346	0.005718	0	0.01597	0.000332	0.000613	0.001376
**TM7**	1.344286	0.11565	2.091743	0.520675	0.063606	0.393575	0.35744	0.591311	0.357406	0.021603	0	0.037262	0.006801	0.002656	0.009238
**Verrucomicrobia**	0.757689	4.298315	0.809707	1.10405	0.081416	0.461321	0.207762	0.397479	0.169616	0.08387	0.115829	0.114068	0.007133	0.006334	0.007273
**WS4**	0	0	0.015571	0	0	0	0.002234	0	0.012115	0.048924	0.043545	0.076046	0.008957	0.014711	0.003341
**WS5**	0	0	0.00519	0	0.002544	0.001613	0.002234	0.014721	0	0	0	0	0	0	0

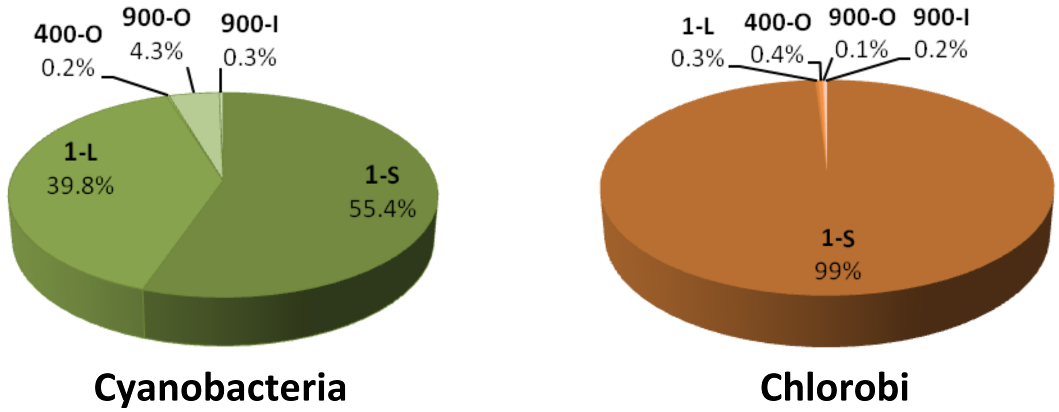

Supplementary Figure S3. Contribution of Cyanobacteria and Chlorobi OTUs to cave ice phototrophic bacterial community in Scarisoara cave ice block. Distribution of the two phyla in 1-S, 1-L, 400-O, 900-O and 900-I ice samples based on their total contribution was considered.

Supplementary Table S2. The most abundant taxa in each ice sample

Sample	Assigned OTU	Taxa	Closest relative [Accession number]	Identity (%)	Origin
1-S	1692	Bacteria; Chlorobi; Chlorobia; Chlorobiales; Chlorobiaceae; Other	Uncultured bacterium [HE616446.1]	99	Lake
	3050	Bacteria; Cyanobacteria; Synechococcophycideae; Pseudanabaenales; Pseudanabaenaceae; Pseudanabaena	Uncultured cyanobacterium [JQ249773.1]	99	Ward Hunt Lake, Nunavut
	30	Bacteria; Proteobacteria; Alphaproteobacteria; Rhizobiales; Bradyrhizobiaceae; Other	*Rhodopseudomonas palustris* [KJ529062.1]	99	Endophytic Bacteria From Deschampsia
	1099	Bacteria; Proteobacteria; Betaproteobacteria; Burkholderiales; Comamonadaceae; Other	Uncultured bacterium [KT752813.1]	97	Glacier Ice
	29	Bacteria; Proteobacteria; Alphaproteobacteria; Rhizobiales; Bradyrhizobiaceae; Bradyrhizobium	*Rhodopseudomonas palustris* [KJ529062.1]	99	Endophytic Bacteria From Deschampsia
	2379	Bacteria; Bacteroidetes; Bacteroidia; Bacteroidales; Other; Other	Uncultured bacterium [KC605963.1]	99	Groundwater
	136	Bacteria; Proteobacteria; Alphaproteobacteria; Rhizobiales; Hyphomicrobiaceae; Devosia	*Devosia humi* [MH463948.1]	99	Low Salinity Lakes On Tibetan Plateau
	81	Bacteria; Verrucomicrobia; Verrucomicrobiae; Verrucomicrobiales; Verrucomicrobiaceae; Prosthecobacter	Uncultured bacterium [KP686975.1]	98	River Water
	109	Bacteria; Cyanobacteria; Oscillatoriophycideae; Oscillatoriales; Phormidiaceae; Phormidium	*Phormidium* sp. CCALA 846 [GQ504034.1]	99	Polar Regions
	111	Bacteria; Proteobacteria; Alphaproteobacteria; Sphingomonadales; Other; Other	*Sphingopyxis* sp. JJ2102 [JX304648.1]	99	Soil
1-L	8	Bacteria; Proteobacteria; Gammaproteobacteria; Pseudomonadales; Pseudomonadaceae; Pseudomonas	Uncultured *Pseudomonas* [HM011849.1]	99	Unfertilized Soil
	100	Bacteria; Proteobacteria; Betaproteobacteria; Burkholderiales; Oxalobacteraceae; Other	*Massilia* sp. strain E4-6 [KY476517.1]	99	Antarctic Soil
	43	Bacteria; Bacteroidetes; Flavobacteriia; Flavobacteriales; Flavobacteriaceae; Flavobacterium	*Flavobacterium sinopsychrotolerans* [MH482266.1]	99	Arctic
	1672	Bacteria; Proteobacteria; Betaproteobacteria; Burkholderiales; Oxalobacteraceae; Other	*Massilia* sp. MY-CA31 [KF747017.1]	98	Cave
	190	Bacteria; Proteobacteria; Betaproteobacteria; Burkholderiales; Comamonadaceae; Other	Uncultured bacterium [JX440994.1]	99	Groundwater

	1087	Bacteria; Firmicutes; Clostridia; Clostridiales; Clostridiaceae; Clostridium	Uncultured *Clostridium* sp. [JX504976.1]	99	Soil
	573	Bacteria; Actinobacteria; Actinobacteria; Actinomycetales; Microbacteriaceae; Cryobacterium	*Cryobacterium roopkundense* [MG952594.1]	99	Cryoconite Sediment
	45	Bacteria; Actinobacteria; Actinobacteria; Actinomycetales; Microbacteriaceae; Other	Uncultured bacterium [EU978795.1]	99	Glacier Ice
	51	Bacteria; Proteobacteria; Alphaproteobacteria; Rhizobiales; Hyphomicrobiaceae; Devosia	*Devosia* sp. B1110_2 [KF295125.1]	99	Muztag Ice Core
	77	Bacteria;Proteobacteria;Betaproteobacteria;Rhodocyclales ;Rhodocyclaceae;Other	Uncultured bacterium [EF590017.2]	98	River
400-O	1	Bacteria; Firmicutes; Bacilli; Bacillales; Paenibacillaceae; Paenibacillus	*Paenibacillus* sp. MN8-13 [JQ396606.1]	99	Arctic Rhizosphere
	40	Bacteria; Bacteroidetes; Bacteroidia; Bacteroidales; Other; Other	Uncultured *Sphingobacteriia* bacterium [KJ650774.1]	99	Kristineberg Tailing Dump
	18	Unassigned; Other; Other; Other; Other; Other	Uncultured bacterium [KT265215.1]	99	Permafrost
	50	Bacteria; Proteobacteria; Gammaproteobacteria; Methylococcales; Crenotrichaceae; Crenothrix	Uncultured bacterium [HM564352.1]	100	Active Layer Soil, High Arctic
	37	Bacteria; Chloroflexi; Anaerolineae; Anaerolineales; Anaerolinaceae; T78	Uncultured *Anaerolineaceae* bacterium [LT841658.1]	99	Anaerobic Digestion
	393	Bacteria; Actinobacteria; Actinobacteria; Actinomycetales; Microbacteriaceae; Other	Uncultured bacterium [JQ684372.1]	98	Permafrost Soil
	873	Bacteria; Actinobacteria; Actinobacteria; Actinomycetales; Cellulomonadaceae; Actinotalea	*Actinotalea fermentans* [KX982788.1]	97	Mangrove Soil
	16	Bacteria; Proteobacteria; Betaproteobacteria; Burkholderiales; Comamonadaceae; Rhodoferax	*Rhodoferax ferrireducens* [KY302292.1]	99	Himalayan Cryoconite Holes
	32	Bacteria; Firmicutes; Clostridia; Clostridiales; Clostridiaceae; Clostridium	Uncultured bacterium [KY690595.1]	99	Sediments From Oligotrophic Andean Lake
	26	Bacteria; Caldiserica; WCHB1-03; Other; Other; Other	Uncultured bacterium [KT265215.1]	99	Permafrost
	42	Bacteria; Actinobacteria; Actinobacteria; Actinomycetales; Intrasporangiaceae; Other	Uncultured *Intrasporangiaceae* bacterium [HE861314.1]	99	Rhizospheric Community Of A Cold Desert
	71	Bacteria; Actinobacteria; Actinobacteria; Actinomycetales; Microbacteriaceae; Other	*Actinobacteridae* bacterium 16-02PB [JX491438.1]	99	Glacier Forefield Soil
	426	Bacteria; Firmicutes; Bacilli; Bacillales; Paenibacillaceae; Paenibacillus	*Paenibacillus* sp. MN8-13 [JQ396606.1]	97	Arctic Rhizosphere

	528	Bacteria; Actinobacteria; Actinobacteria; Actinomycetales; Cellulomonadaceae; Actinotalea	Uncultured bacterium [JQ684378.1]	98	Permafrost Soil Samples From Kunlun
900-O	1645	Bacteria; Actinobacteria; Actinobacteria; Actinomycetales; Microbacteriaceae; Cryobacterium	Uncultured *Cryobacterium* sp. [EU852130.1]	96	Soil India: Roopkund Glacier
	974	Bacteria; Actinobacteria; Actinobacteria; Actinomycetales; Nocardioidaceae; Aeromicrobium	Uncultured bacterium [KM128408.1]	99	Water Lake
	309	Bacteria; Firmicutes; Clostridia; Clostridiales; Clostridiaceae; Clostridium	Uncultured *Saccharofermentans* sp. [LT842409.1]	99	Anaerobic Digestion
	1537	Bacteria; Firmicutes; Clostridia; Clostridiales; Christensenellaceae; Other	Uncultured bacterium [MG855118.1]	99	Rice Straw Anaerobic Digester
	1423	Bacteria; Actinobacteria; Actinobacteria; Actinomycetales; Cellulomonadaceae; Cellulomonas	Uncultured bacterium [KY190744.1]	96	Hydrocarbon Polluted Soil From Antarctic Station
	1087	Unassigned;Other;Other;Other;Other;Other	Uncultured bacterium [DQ088235.1]	93	Shuangcheng Moat Sediment
	1182	Bacteria; Proteobacteria; Gammaproteobacteria; Enterobacteriales; Enterobacteriaceae; Other	*Citrobacter freundii* [KT156820.1]	99	Gut Fly
	784	Bacteria; Firmicutes; Bacilli; Bacillales; Paenibacillaceae; Paenibacillus	*Paenibacillus* sp. EA13 [JN819628.1]	93	Soils
	10	Bacteria; Actinobacteria; Actinobacteria; Actinomycetales; Microbacteriaceae; Other	*Salinibacterium sp.* [KY476565.1]	99	Antarctic Soils
	88	Bacteria; Actinobacteria; Actinobacteria; Actinomycetales; Mycobacteriaceae; Mycobacterium	*Mycobacterium sp.* [MH298485.1]	99	Pinus Nigra Trunk
900-I	86	Bacteria; Actinobacteria; Thermoleophilia; Solirubrobacterales; Other; Other	Uncultured bacterium [KY190671.1]	99	Hydrocarbon Polluted Soil From Antarctic Station
	5	Bacteria; Actinobacteria; Actinobacteria; Actinomycetales; Cellulomonadaceae; Actinotalea	Uncultured bacterium [JQ684372.1]	99	Permafrost Soil Samples From Kunlun
	1280	Bacteria; Caldiserica; WCHB1-03; Other; Other; Other	Uncultured bacterium [KT265215.1]	98	Permafrost
	1402	Unassigned; Other; Other; Other; Other; Other	Uncultured bacterium [FJ770025.1]	99	Antarctic Soils
	1275	Bacteria; Actinobacteria; Actinobacteria; Actinomycetales; Nocardioidaceae; Aeromicrobium	*Nocardioides* sp. [KY386444.1]	96	Antarctic Soils
	315	Bacteria;Actinobacteria;Acidimicrobiia;Acidimicrobiales; EB1017;Other	Uncultured bacterium [KT793092.1]	97	Peatland Sample

Supplementary Table S3. Geochemical properties of ice samples

Sample	pH	EC (μS cm^{-1})	TOC (mg L^{-1})	TN (mg L^{-1})
**1-S**	7.48	124.2	33.38 ± 0.48	2.15 ± 0.03
**1-L**	7.57	65.1	11.28 ± 0.45	0.53 ± 0.01
**400-O**	7.45	61.6	29.96 ± 0.95	2.23 ± 0.03
**900-O**	7.87	17.3	6.48 ± 0.30	0.62 ± 0.01
**900-I**	8.03	15.0	3.03 ± 0.12	0.64 ± 0.09

The pH, and electrical conductivity (EC) values of melted ice samples 1-S, 1-L, 400-O, 900-O and 900-I were measured at 22˚C. The average values and standard deviation of total organic carbon (TOC) and total nitrogen (TN) contents of ice samples were calculated from three different measurements (adapted from Itcus et al, 2016[33]).
